# Ethosome-Based Transdermal Drug Delivery: Its Structural Components, Preparation Techniques, and Therapeutic Applications Across Metabolic, Chronic, and Oncological Conditions

**DOI:** 10.3390/pharmaceutics17050583

**Published:** 2025-04-29

**Authors:** Rashed M. Almuqbil, Bandar Aldhubiab

**Affiliations:** Department of Pharmaceutical Sciences, College of Clinical Pharmacy, King Faisal University, Al-Ahsa 31982, Saudi Arabia; baldhubiab@kfu.edu.sa

**Keywords:** transdermal drug delivery, ethosomes, metabolic diseases, chronic diseases, nanomedicine

## Abstract

Transdermal drug delivery systems (TDDSs) provide a non-invasive alternative to oral and parenteral routes, delivering drugs into the bloodstream while avoiding gastrointestinal degradation and first-pass metabolism. Despite benefits like enhanced bioavailability and patient compliance, the stratum corneum limits drug permeation. Ethosomes overcome the stratum corneum barrier with superior flexibility and permeability compared to liposomes. Ethanol disrupts the skin’s lipid bilayer, enabling deep penetration and efficient drug delivery. Ethosomes offer high entrapment efficiency and stability, delivering both hydrophilic and lipophilic drugs. However, challenges like stability optimization and clinical translation persist. This review examines the structural components, preparation methods, and therapeutic applications of ethosomes in metabolic and chronic diseases, including diabetes, cardiovascular diseases, neurodegenerative disorders, arthritis, and cancers. Moreover, it highlights the potential of ethosomes to revolutionize TDDSs for managing chronic and metabolic diseases, providing a foundation for further research and clinical development.

## 1. Introduction

Transdermal drug delivery systems (TDDSs) are an innovative and patient-friendly approach for drug delivery and have several benefits over conventional oral and parenteral drug delivery systems. These systems are designed to transport medication through the skin membrane and into the bloodstream, maintaining constant drug levels for an extended period. They deliver drugs directly into the bloodstream, bypassing gastrointestinal degradation and first-pass metabolism by the liver [1]. Over the past few decades, TDDSs have attracted the attention of the pharmaceutical industry and clinical practice due to their capacity to improve patient compliance, lower frequency of dosing, and, very importantly, increase bioavailability. Although the large surface area of the skin allows drugs to be absorbed somewhat easily, its primary purpose is to act as a protective barrier, which makes drug access challenging. To overcome this hurdle, pharmaceutical research has successfully developed many TDDSs for clinical practice.

For instance, transdermal patches like nitroglycerin for angina, fentanyl for pain management, scopolamine for motion sickness, estradiol for hormone replacement therapy, and clonidine for hypertension are among those [2,3]. TDDSs, including patches, gels, and vesicular systems like ethosomes, offer flexible administration; for instance, removing a patch or discontinuing application can rapidly terminate drug delivery, unlike oral administration, where cessation is less possible. At present, TDDSs are widely used in the management of various physiological conditions, like pain management, hormone therapy, neurological diseases, vaccine delivery, and cardiovascular diseases [4]. This progress was made possible by TDDSs, which use advanced technologies, such as nanoparticles, microneedles, and vesicular systems like ethosomes, to enhance drug penetration through the skin by improving delivery efficiency and enabling deeper tissue access [5].

The selective permeability of the skin significantly limits TDDS application, despite its various advantages. The stratum corneum layer, the top layer of the epidermis, acts as a protective barrier by allowing only low molecular weight compounds to pass through it [6]. The transport of molecules into and through the skin is influenced by drug parameters such as the molecular mass, melting point, log P, pKa, and dose [7]. Thus, only molecules with a mass of less than 500 daltons and partition coefficients within a favorable range can effectively permeate the skin [8]. This limitation poses a considerable challenge to the effective delivery of specific therapeutic agents. It is also hard to obtain quick plasma levels of drugs with conventional TDDSs because drugs passively diffuse through the skin. This makes it hard to treat acute pain or provide emergency care. Moreover, the absorption efficacy and treatment outcomes of TDDSs are also affected by age, skin hydration, the place of drug administration, the temperature of the skin, skin disorders, wounds, and the excessive sweating of individuals [9]. The limited drug-loading capacity of certain TDDSs, such as patches, can restrict the amount of drug delivered, often necessitating multiple applications or combinations with other therapies to achieve optimal therapeutic outcomes [10]. Most TDDS systems allow the inclusion of a penetration enhancer, which may aggravate the skin of patients with sensitive skin [11]. Specific surfactants and fatty acids incorporated into transdermal drug delivery system (TDDS) formulations, along with ethanol in ethosomes, function as penetration enhancers by disrupting the stratum corneum’s lipid structure, potentially leading to mild irritation or the compromising of the skin’s outermost barrier [12]. This can cause allergic dermatitis and make patients less likely to follow through with their therapy. Lastly, the cost of such technological dosage forms comes from the use of specialized materials, and complex regulatory challenges for bioequivalence, safety, and stability that altogether encompass cost-based challenges toward TDDS use [13].

Nanoparticle technology-based drug delivery has gained significant recognition in the pharmaceutical industry for its ability to enhance solubility, stability, bioavailability, and targeted drug delivery. Various nanocarriers, such as liposomes, solid lipid nanoparticles, polymeric nanoparticles, and nanostructured lipid carriers, have undergone thorough research and investigation to overcome conventional limitations and enhance skin delivery [14,15]. These systems improve drug penetration, protect active ingredients from degradation, enhance the surface area for absorption, offer controlled and sustained release, and improve penetration through the skin [10]. Ethosomes are one of the most promising vesicular nanocarriers developed for transdermal medication administration. Ethosomes are a type of lipid-based vesicle that was initially described in 1997 by Touitou et al. [16]. They consist of phospholipids, a high concentration of ethanol (20–45%), and water. Ethosomes have more flexibility and adaptability than typical liposomes, which have restricted penetration because of their rigid bilayer structure [17], enabling them to pass the skin’s stratum corneum barrier effectively [18]. The higher concentration of ethanol makes ethosomes unique, as it improves permeability by loosening tight intercellular connections and thus enabling deep drug penetration by altering the lipid layer of the skin. Moreover, ethanol imparts a negative charge to the ethosome surface [19], increases vesicle fluidity, and enables them to squeeze through narrow intercellular spaces [20]. Ethosomes are very good at delivering hydrophilic and lipophilic drugs because of this unique structural advantage, increasing bioavailability and therapeutic efficacy. Compared to traditional transdermal carriers, ethosomes offer superior encapsulation efficiency, prolonged drug retention, low irritation, the capability to carry active components deeper into the skin, reduced systemic side effects, and improved patient compliance, making them an advanced and viable alternative to conventional transdermal drug delivery systems [21].

While ethosomes, as a transdermal drug delivery system, are designed to deliver drugs into systemic circulation, recent research has expanded their applications beyond dermatological conditions to include systemic treatments for a wide range of diseases, such as cancer, diabetes, and chronic pain. Studies have shown that ethosomal transdermal delivery is effective in treating and managing metabolic and chronic diseases. This review aims to comprehensively analyze ethosome-based transdermal drug delivery, focusing on its structural components, preparation techniques, and therapeutic applications in metabolic and chronic diseases. We have given special attention to significant metabolic and chronic diseases such as cancer, diabetes, arthritis, and neurological and cardiovascular diseases. This updated review will be a valuable resource for researchers, clinicians, and pharmaceutical scientists seeking to understand the latest advancements of ethosome-based drug delivery for chronic and metabolic diseases.

## 2. Ethosomes

Ethosomes are advanced lipid-based vesicular delivery systems that, unlike conventional liposomes, incorporate a high ethanol content, enhancing their flexibility and transdermal penetration capabilities. Ethosomes are primarily used in pharmaceutical applications to enhance transdermal drug delivery for both localized and systemic therapeutic effects, and they are also explored in cosmetics to improve the penetration of active ingredients into the skin’s upper layers. Due to the higher ethanol content in ethosomes compared to other forms of vesicular systems like liposomes, which act as a penetration enhancer, ethosomes affect the intracellular level in the stratum corneum [22]. They are believed to disrupt the thick stratum corneum and deliver the drugs into the deep tissue and the site of action. Ethosomes were developed to address the challenge of poor skin penetration in transdermal drug delivery, as earlier systems struggled to deliver active compounds through the stratum corneum effectively. Scientists have developed various other nanoforms to overcome this issue. For instance, first, they developed transfersomes and niosomes. Transfersomes are ultra-deformable vesicles that penetrate deep into the skin by squeezing through narrow pores due to their high flexibility, using osmotic gradients to enhance transdermal drug delivery [23]. Niosomes are non-ionic surfactant-based vesicles used for drug delivery, similar to liposomes, but are more stable and cost-effective [24,25]. Scientists formulated ethosomes later, leading to a breakthrough in TDDSs.

## 3. Advantages of Ethosomes

Ethosomes offer definite advantages over traditional transdermal drug delivery systems due to their unique composition. Their high ethanol content enhances skin permeability by fluidizing the stratum corneum, allowing efficient delivery of both hydrophilic and lipophilic drugs. Compared to liposomes and niosomes, ethosomes exhibit greater flexibility, enabling deeper penetration through skin barriers. They provide high entrapment efficiency and stability, minimizing vesicle aggregation. Ethosomes are safe, cost-effective, and suitable for immediate commercialization, requiring no invasive techniques, which enhances patient compliance and broadens their therapeutic and cosmetic applications [26,27,28].

## 4. Types of Ethosomes

Ethosomes are categorized into several types, including classical ethosomes, binary ethosomes, transethosomes, composite phospholipid ethosomes, and actively targeted ethosomes, each designed to optimize transdermal drug delivery.

**Figure 1:**
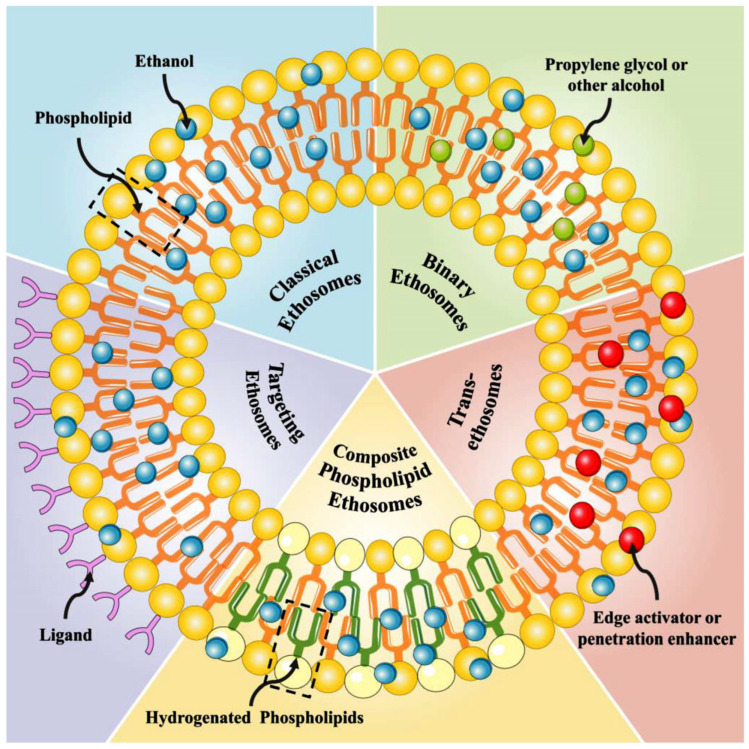
Structure of ethosomes. Reproduced from Ref. [30].

Classical ethosomes are lipid-based nanocarriers of phospholipids, ethanol (20–45%), and water. They offer high permeability and ease of formulation compared to other varieties of ethosomes [18,29]. Classical ethosomes offer better skin stability and compatibility, whereas other forms, such as binary ethosomes, needs the addition of penetration enhancers or transethosomes, which incorporate surfactants that may cause irritation. The classical form of ethosomes is superior to polymer-based ethosomes in terms of the cost of manufacturing. Different types of ethosomes are available, such as classical ethosomes, binary ethosomes, and transethosomes (Figure 1).

Binary ethosomes are an advanced version of classical ethosomes, in which another type of alcohol is incorporated as a penetration enhancer [31]. For example, propylene glycol or isopropyl alcohol will be found in these types, in addition to ethanol and phospholipids. As a second alcohol is added into the vesicles; it helps the drug carrier to pass through the stratum corneum by fluidizing it. Hence, the use of two types of alcohol gives synergetic action over normal ethosomes and provides superior skin permeability and drug retention. Compared to transethosomes, which contain surfactants that can potentially irritate the skin, binary ethosomes offer improved skin biocompatibility whilst still facilitating effective drug penetration. While ethanol in classical ethosomes contributes to vesicle stability by reducing aggregation, the additional alcohol in binary ethosomes further optimizes vesicle fluidity and stability, potentially minimizing aggregation under specific formulation conditions. Together with all these advantages, binary ethosomes are preferred for both hydrophilic and lipophilic drugs, not only for dermatological applications but also for systemic applications [32].

Transethosomes are a novel form of ethosomes that were first described by Song et al. in 2012 [33]. These kinds of ethosomes are similar to the classic type of ethosomes, but they incorporate surfactants or edge activators (e.g., Tween 80, Span 80, or sodium cholate) in addition to ethanol, phospholipids, and water. They have been designed to combine the advantages of classical ethosomes and deformable liposomes (transfersomes) as a single delivery system to produce transethosomes. The surfactants used in these vesicles enhance their flexibility to pass through the skin barrier. Transethosomes are recognized for their higher flexibility and permeability compared to classical and binary ethosomes. This makes them particularly effective for the delivery of large molecules like peptides and proteins. Studies have shown that they can entrap molecules of sizes up to 325 kDa [34]. Transethosomes offer better stability and lower vesicle aggregation than classical ethosomes due to the presence of surfactants that prevent vesicle fusion [35].

There are two more types of ethosomes which are considered minor ethosomes, such as composite phospholipid ethosomes and actively targeted ethosomes, otherwise called functionalized targeted ethosomes. Composite phospholipid ethosomes are specially designed to prevent unsaturated phospholipid oxidation. Studies have pointed out that composite phospholipid ethosomes offer superior drug entrapment and enhanced topical delivery compared to classical ethosomes [36]. Functionalized targeted ethosomes are designed to carry drugs to specifically targeted areas such as acne and some skin infections. Functionalized ethosomes have the advantages of increased stability, improved transdermal performance, an extended, prolonged drug release profile, and site-specific delivery, due to their functional materials [37]. Nowadays, many medical conditions are actively targeted by functionalized ethosome formulations [38,39,40].

## 5. Mechanism of Action of Ethosomes

**Figure 2:**
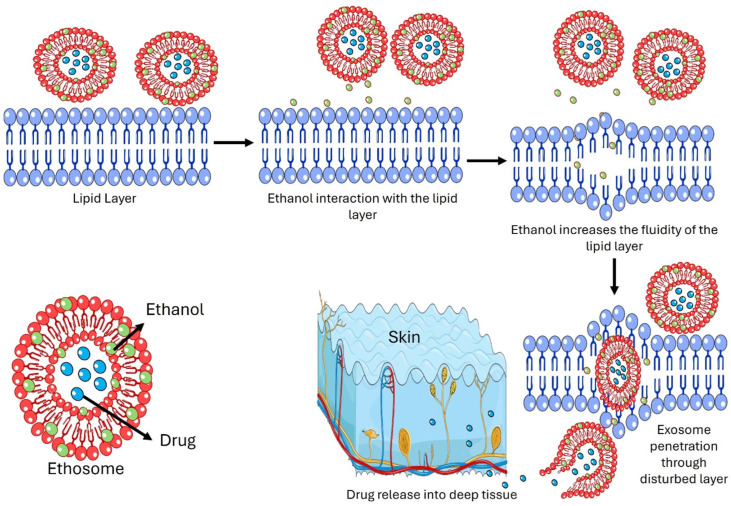
Mechanism of action of ethosomes as transdermal drug delivery.

The mode of action and delivery from ethosome formulation is due to an interaction between ethanol, lipid layers, and vesicles (Figure 2). In general, the mechanism of ethosomes can be broadly divided into two stages. One stage is called the ‘ethanol effect’, in which ethanol directly interacts with the lipid bilayer hydrophilic head. By that, it reduces the transition temperature, increases lipid layer fluidity, and reduces layer density. They act in this stage by (1) drug solubilizing enhancement, in which ethanol acts as a penetration enhancer, increasing the solubility of the drug in the lipid bilayer of the ethosomes. This ensures the efficient encapsulation of both hydrophilic and hydrophobic drugs; (2) interaction with the stratum corneum, in which the high ethanol content disrupts the lipid organization of the stratum corneum, the outermost layer of the skin. In addition, ethanol fluidizes the lipids, reducing the barrier function and allowing ethosomes to penetrate deeper into the skin; (3) vesicle flexibility and deformability, in which ethosomes are highly flexible due to the presence of ethanol, which reduces the rigidity of the phospholipid bilayer, which therefore enables it to squeeze through the narrow intercellular spaces of the stratum corneum, a process known as vesicle-driven transdermal penetration. The second stage, termed the ‘ethosome effect’, involves the malleability and fusion of ethosomes with the skin’s lipid layers, facilitating drug release. They act in this stage by (1) fusing with skin lipids, in which ethosomes can fuse with the lipids in the stratum corneum and deeper skin layers, releasing the drug directly into the skin; (2) deep penetration into the skin, in which ethosomes can penetrate deeper into the dermis and even reach systemic circulation if designed for transdermal delivery; and (3) controlled and sustained release, in which the ethosome inside the skin acts as a reservoir for the controlled and sustained release of the drug in order to keep a steady state level of the drug in blood circulation.

## 6. Preparation of Ethosomes

### 6.1. Cold Method

**Figure 3:**
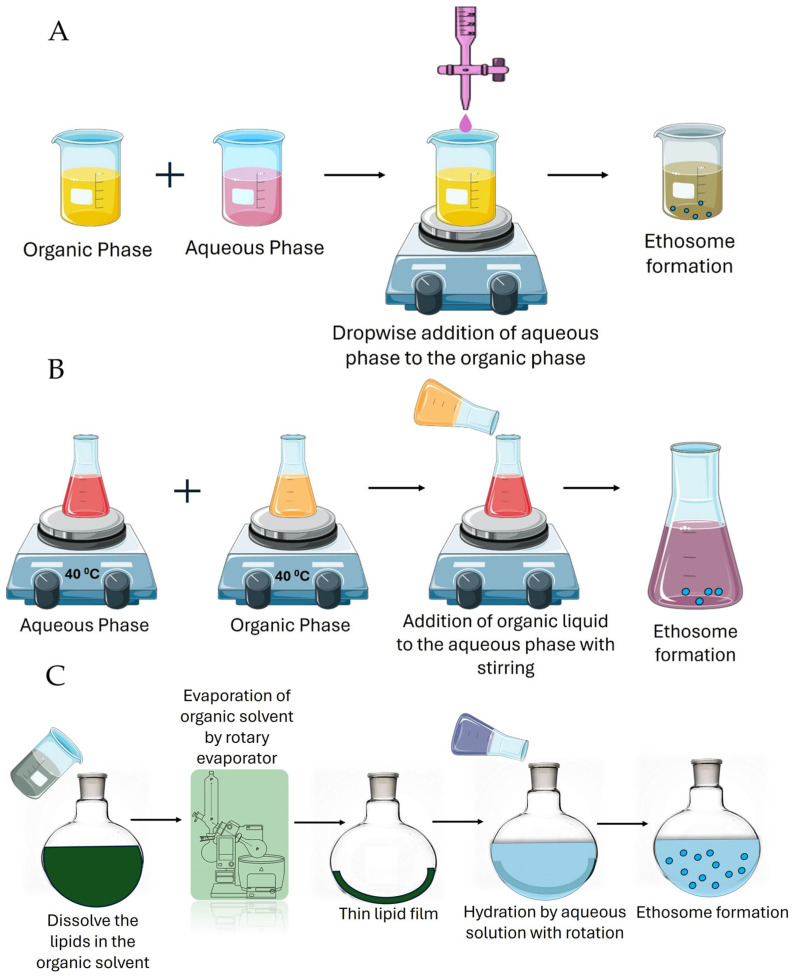
The method of preparation of ethosomes. (**A**) The cold method; (**B**) the hot method; and (**C**) the thin film method. The image was created using pictures from Servier Medical Art, by Servier (http://smart.servier.com, accessed on 5 March 2025) through creativecommons.org/licenses/by/4.0/.

The cold method is the simplest and most common method of preparing ethosomes (Figure 3A). In this method, the organic and aqueous phases are made separately. At 25 °C, with vigorous agitation, ethanol 20% to 45% (*w*/*w*) is solubilized into a mixture of phospholipids, pharmaceutical agents, and other lipid constituents. The container is then heated to 30 °C. This method, commonly termed the ’cold approach’, is widely employed. Water is preheated to 30 °C in a separate vessel and is gradually incorporated into the initial mixture with continuous stirring. Vesicle formation initiates after approximately 5 min of mixing. Maintaining the resulting vesicles at a low temperature is critical. Optionally, the resulting ethosomal dispersion can be sonicated for a specified duration to further reduce the vesicle size and enhance uniformity [18,28].

### 6.2. Hot Method

The hot method of ethosome preparation involves dissolving the phospholipids in ethanol at an elevated temperature and then adding water to the mixture while continuously stirring. Initially, the phospholipids are dissolved in ethanol at 40–60 °C with constant stirring (Figure 3B). Then, a heated aqueous phase (e.g., water or a drug solution) at the same temperature is slowly added to the ethanolic phase under constant stirring. The mixture is mixed at a controlled speed to form the ethosome vesicle. The vesicle formed may be sonicated to achieve uniform-sized vesicles for 5 min or for the desired time to achieve the optimum size. This method is simple and efficient, especially for thermally stable drugs, and ensures good ethosome formation with high encapsulation efficiency. However, it may not be suitable for heat-sensitive drugs [41].

### 6.3. Thin Film Method

This method of preparation of ethosomes is suitable for the encapsulation of both hydrophilic and lipophilic drugs. It gives better control over the vesicle size and drug loading with better entrapment efficacy compared to other conventional methods (Figure 3C). Firstly, phospholipids are dissolved in ethanol and an organic solvent, and then a rotary evaporator is used to evaporate the solvent to make a thin film. The film inside the flask is hydrated with PBS or water that contains the drug with constant stirring at a suitable temperature. Ethanol is added to the hydrated vesicles to enhance membrane fluidity and penetration. This method is ideal in transdermal applications [42].

## 7. Application of Ethosomes in Disease Management

Ethosomes have revolutionized nanotechnology-based drug delivery, offering a large opportunity to deliver various drugs into blood circulation in a non-invasive way. Hence, many studies have been published in the literature that highlight their role in disease management. The adaptable nature of ethosomes enables the effective delivery of diverse therapeutic agents, positioning ethosomes as a promising tool for managing a vast array of diseases. In dermatology, ethosomes are used to treat psoriasis [43], eczema [44], acne [45], and vitiligo [46], delivering corticosteroids, retinoids, or depigmenting agents like hydroquinone with superior skin retention and minimal systemic effects. In infectious diseases, ethosomal formulations have proven successful in treating fungal infections like candidiasis with clotrimazole [47], cutaneous infections with antibacterials like erythromycin [48], and managing herpes simplex and HIV with antiretroviral acyclovir [49]. Ethosomes have offered enhanced corneal delivery, serving ocular diseases such as glaucoma with timolol [50]. They offered excellent alternatives to oral drug administrations to treat gastrointestinal disorders, such as vomiting, with rectal ethosome domperidone gel [51]; regenerative applications include wound healing [52], the treatment of viral infections like hepatitis B [53], and vaccine delivery [54].

## 8. Therapeutic Applications in Selected Metabolic and Chronic Diseases

Metabolic and chronic diseases represent a significant global health challenge, often interconnected through complex physiological mechanisms. Metabolic diseases are caused by the body’s inability to metabolize and utilize nutrition, protein, and fats, often due to a lack of hormones or enzymes. These conditions can be formed due to sedentary lifestyles, environmental conditions, or genetic conditions [55,56]. These changes in the body can disrupt homeostasis and energy utilization and cause conditions such as diabetes mellitus, obesity, etc. [57]. At the same time, chronic diseases are conditions that last a long time in the body, progress slowly, and cannot be fully treated, which need constant monitoring and treatments for life. Cancers, cardiovascular issues, COPD, chronic kidney disease, etc., are well-known chronic diseases [58]. The link between metabolic and chronic diseases is often unclear because metabolic dysfunction can be a precursor to chronic conditions [59]. The close relationship between obesity and diabetes, as well as diabetes and cardiovascular diseases, metabolic syndromes, kidney diseases, etc., is well established [60].

Non-invasive drug delivery plays an essential role in the delivery of pharmaceuticals for many disease conditions. Many studies have proved that they are excellent in managing many chronic and metabolic conditions [61]. Non-invasive drug delivery systems, such as transdermal ethosomes, offer advantages over invasive methods or other routes of administration, including controlled drug release, the maintenance of steady-state drug levels, the avoidance of first-pass metabolism, and improved patient compliance due to their non-invasive nature. Transdermal systems can deliver the drug in steady-state levels in dosage forms such as patches and microneedles. This makes it very important in the management of chronic conditions, which need constant drug delivery, for instance, in diabetes mellitus and blood pressure management. Another advantage is the avoidance of drug degradation from stomach acids, such as insulin, which can be easily achieved by transdermal delivery [62]. Various formulation technologies can achieve this, but the advancement of nanotechnology has revolutionized this delivery system. Liposomes, nanoemulsions, lipid nanoparticles, nanostructured lipid carriers, nanomicelles, dendrimers, electrospun nanofiber, niosomes, and transferosomes are the different categories of nanoformulation applied in transdermal drug delivery systems [63,64,65]. However, above all, ethosomes hold superior characteristics, especially in dermal drug delivery. Through the enhanced penetration of active ingredients into the skin, ethosomes are superior compared to other nanoformulations. The application of ethosomes in mitigating metabolic and chronic diseases is listed in Table 1.

### 8.1. Management of Diabetic Mellitus

Diabetes mellitus, a chronic metabolic disorder, impairs the body’s ability to regulate blood glucose, impacting multiple organs. According to the updated available data, diabetes accounts for 67.9 million disability-adjusted life years (DALYs) globally, with 52.2% of type 2 diabetes DALYs linked to a high BMI [89]. Current drug delivery systems, such as oral formulations, face challenges like low bioavailability and drug degradation in the stomach, which transdermal ethosomal systems aim to overcome by enhancing drug stability and delivery efficiency [90].

Glimepiride is one of the best anti-diabetic oral drugs in clinical practices, but it has many formulation issues. Glimepiride is present in two polymorphic forms (GLIM I and II). As per established studies, GLIM form II is roughly 3.5 times more soluble and delivers twice as much drug as GLIM form I within the body’s typical pH range. This leads to greater insulin secretion and lower blood glucose levels with GLIM II and can potentially increase risks for patients, including the possibility of hypoglycemia. Hence, selecting the correct isomer form of the drug is critical for the safety of the patient [91]. The adaptation of transdermal drug delivery for glimepiride may overcome these challenges, and at the same time, it can deliver a well-controlled, steady-state level of drug in the blood for optimum glycemic control. Tiwari and colleagues formulated a glimepiride ethosomal formulation to evaluate the effect of alcohol and phospholipid on the topical delivery of the drug. Formulations were made with alcohol, soya lecithin, and propylene glycol in different proportions. The final formulations were evaluated for their entrapment efficiency, zeta potential, turbidity, size of the vesicle, etc. They found the vesicle size to be between 22 and 105 nm. Entrapment efficacy was achieved at a maximum of 58.4 ± 0.3% for both the alcohol and isopropyl alcohol formulations. The interaction of the ethosome formulation (drug, polymers, and excipients) was revealed by the results of FT-IR, whereas the turbidity and particle size were determined by the zeta potential. In vitro drug release through the egg membrane revealed that the ethosome formulation showed up to 49.01 ± 0.03% release for 24 h, together with efficient stability at room temperature [92].

**Figure 4:**
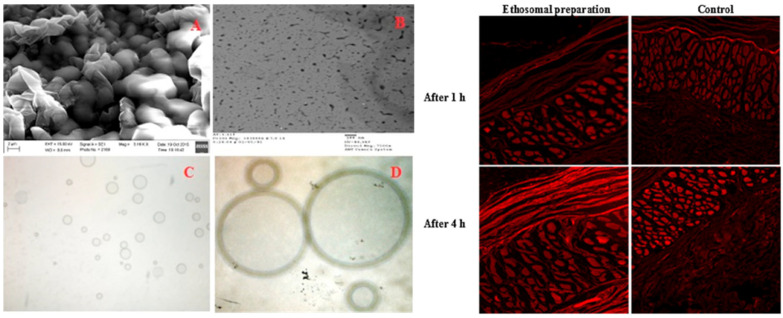
Scanning (**A**) and transmission electron (**B**) microscopic images at 100× (**C**) and 400× (**D**) magnification power of glimepiride optimized in ethosomal nano-vesicles (**left**); confocal laser microscope images for rat skin layers after application of fluorescence-labeled ethosomes films and rhodamine-loaded film at different time points (**right**). Reproduced with permission from reference [93]. Copyright 2016, Elsevier B.V.

Ahmed and colleagues have developed an ethosomal formulation of glimepiride for transdermal application to minimize the side effects and facilitate long drug release. They made formulations with significant optimization on vesicle size, entrapment efficiency, and vesicle flexibility. In order to achieve this, the alcohol percentage, propylene glycol, cholesterol, and phospholipid content were adjusted in the formulation using the Draper–Lin small composite design. The ideal ethosomal formulation showed a flexibility of 54.03%, an entrapment effectiveness of 97.12%, and a vesicle size of 61 nm. The ethosomes were then included in transdermal films and studied for ex vivo permeation investigations on rat skin, which showed that the ethosomal films greatly improved drug penetration as compared to films including pure glimepiride. Confocal laser imaging confirmed the uniform penetration of the fluorescence-labeled ethosomal formulation across the stratum corneum, viable epidermis, and dermis of rat skin, with a higher fluorescence intensity compared to the control, indicating the enhanced distribution of the ethosomal carrier and supporting improved glimepiride delivery. In vivo experiments on human volunteers suggested reduced adverse effects, which showed prolonged drug release and lower maximum plasma concentrations compared to oral treatment. The ethosomal transdermal films showed promise since pharmacokinetic parameters like C_max_ and AUC parameters indicated continuous drug levels over 12 h compared to oral tablets (Figure 4). For weakly water-soluble medications like glimepiride, the study emphasizes the potential of ethosomes in improving transdermal drug delivery [93].

**Figure 5:**
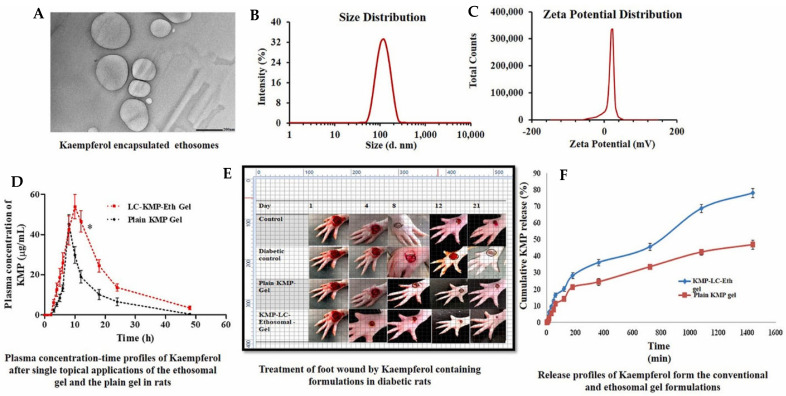
Hybrid lecithin–chitosan kaempferol ethosomes were prepared to treat diabetic foot ulcers. The surface morphology of the optimized ethosomes was captured with a transmission electron microscope (**A**) at magnifications of 10K×; the vesicular size of the nanoformulation was found to be 186.8 nm (**B**); the particle was recorded with a high zeta potential of 31.9 mV (**C**); the plasma concentration–time profile of ethosome formulation after a single topical application of the optimal LC-KMP-Eth gel (**D**) was recorded; significantly improved wound healing on a foot (**E**) was captured; and in vitro release profiling of the formulation (**F**) also were recorded. Images were taken with permission from the reference [94]. Copyright 2024, Elsevier B.V.

Apart from diabetic management, ethosomal formulations are also found to be excellent for diabetic foot ulcer management. Raghav and colleagues used the formulation of ethosome using kaempferol, a natural product that has a high antioxidant potential [94]. The study explored hybrid lecithin–chitosan (LC) ethosomes as a topical delivery system for kaempferol to treat diabetic foot ulcers. The formulation aimed to enhance encapsulation efficiency and controlled release. The optimized ethosomes, which contained 253 mg lecithin, 0.25 mg/mL chitosan (to improve permeation), and 30% ethanol, achieved a vesicular size of 182.5 nm, a polydispersity index of 0.285, a zeta potential of 31.6 mV, and an impressive entrapment efficiency of 95.3%. These were incorporated into a 1% Carbopol 934 gel, exhibiting a pH of 6.8 ± 0.5 and a viscosity of 6.8 ± 1.2 Pa·s. In vitro studies showed the ethosome gel released 90.2% kaempferol over 1440 min, significantly higher (*p* < 0.05) than the 46.9% from plain kaempferol gel, following a Fickian diffusion mechanism (R^2^ = 0.988, n < 0.45). Stability tests confirmed better retention of properties (VS: 198.7 ± 8.4 nm, EE%: 91.5 ± 3.5%) at 4 °C over three months compared to 25 °C. In vivo studies conducted in Wistar rats showed superior wound healing in rats with the ethosomal gel, reducing swelling by day 4 and achieving a wound area of 2.83 ± 0.56 mm^2^ by day 21 compared to 24.6 ± 0.66 mm^2^ in the diabetic control. Pharmacokinetic analysis revealed an AUC_0–48_ of 854.9 µg·h/mL for the formulation against 428.91 ± 46.81 μg/ML·h for plain gel, with a 1.7-fold increased half-life. Antimicrobial tests showed the formulation with larger inhibition zones (15.64 ± 1.20–17.98 ± 0.89 mm) against *Staphylococcus aureus* strains compared to pure kaempferol (10.32 ± 1.85–12.89 ± 1.65 mm). Histopathology confirmed enhanced re-epithelialization and granulation (Figure 5). These findings highlight the lecithin–chitosan–kaempferol–ethosome gel as a promising diabetic foot ulcer treatment, leveraging improved drug delivery and therapeutic efficacy.

### 8.2. Management of Cardiovascular Diseases

Cardiovascular diseases are a group of diseases, including coronary heart disease, hypertension, heart failure, arrhythmia, stroke, peripheral artery disease, and coronary artery disease, which fall under this category [95,96,97]. Studies have shown that cardiovascular diseases remain the most common cause of death in Europe, with over 3 million deaths per year [98]. Metabolic syndrome is a major contributing factor to the rise in heart diseases [99]. Oral and parenteral medications face challenges like low bioavailability and inconsistent drug levels, contributing to conditions such as hypertension fluctuations [100,101]. Ethosomal transdermal delivery offers a promising solution by improving drug bioavailability and enabling controlled release.

Carvedilol is a non-selective beta-blocker that possesses no selective alpha-1 blocking properties. It is frequently used in the management of hypertension, heart failure, and left ventricular dysfunction after a heart attack [102]. It works by blocking beta-adrenergic receptors, which reduces heart rate and myocardial contractility, eventually resulting in decreased oxygen demand and enhanced cardiac efficiency. In contrast to certain beta-blockers, carvedilol possesses antioxidant properties that could offer extra cardiovascular benefits [103]. It is generally well accepted, though frequent side effects may consist of dizziness, fatigue, low blood pressure, and a slow heart rate. The oral administration of carvedilol is well tolerated, but its bioavailability is limited to only 25%, and the half-life is about 6 h due to first-pass metabolism [104]. It has been selected as an excellent choice of drug to be formulated as ethosomes since its properties include low molecular weight, ideal partition coefficient, and poor bioavailability.

Amarachinta et al. published a research study that aimed to optimize carvedilol-loaded ethosomes and the incorporation of synthesized ethosomes into hydrogels for the transdermal delivery of carvedilol [105]. They found that optimized ethosomes have a vesicle size of 130 ± 1.72 nm, an entrapment efficiency of 99.12 ± 2.96%, a cumulative drug release of 97.89 ± 3.7%, a zeta potential of −31 ± 1.8 mV, and a polydispersity index of 0.230 ± 0.03. After incorporating ethosomes into the hydrogels, the formulation showed sustained release for up to 72 h, higher skin permeation, and better retention compared to the free drug-loaded hydrogel. In vivo studies in rats demonstrated gradual blood pressure reduction over 24 h, suggesting that ethosomal hydrogels are an effective transdermal delivery system for prolonged anti-hypertensive effects.

**Figure 6:**
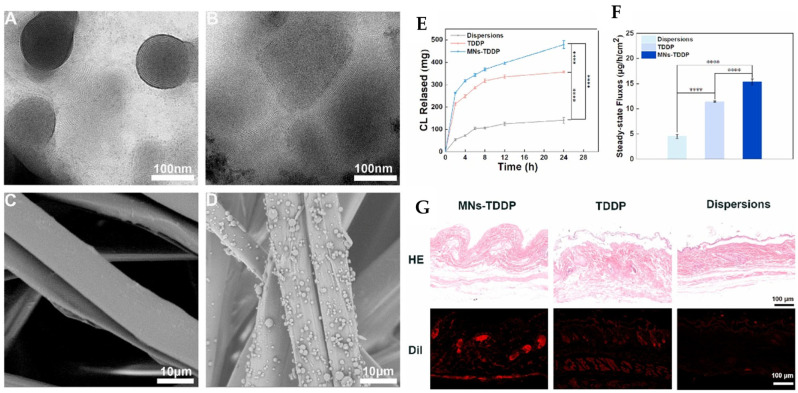
The bioavailability of carvedilol was improved by formulating binary ethosome-based transdermal patches. Transmission electron microscopy pictures of binary ethosomes (**A**), carvedilol binary ethosomes (**B**), scanning electron microscopy of silk fibroin matrices (**C**), and transdermal drug delivery patches (**D**) were recorded. The in vitro transdermal data were obtained as a cumulative drug release curve (**E**), a steady-state release flux (**F**), and a skin section H&E stain, and fluorescence images (**G**) were recorded. **** *p* < 0.0001. The images were taken with permission from the reference [106]. Copyright 2022, Elsevier B.V.

Later, Di Jiang et al. introduced an innovative transdermal drug delivery system combining binary ethosomes and metal microneedles to enhance the bioavailability of carvedilol [106]. The formulations were optimized using the Box–Behnken design, achieving a particle size of 506.5 ± 50.85 nm, a zeta potential of 11.30 ± 1.00 mV, an encapsulation efficiency of 73.50 ± 2.51%, and a 24 h cumulative drug release of 361.04 ± 38.91 µg with an optimal formula of 0.10 g lecithin, 0.01 g octadecylamine, 20% *v*/*v* ethanol, and 10% *v*/*v* propanediol. These ethosome formulations were incorporated into TDDP using a silk fibroin matrix and polyvinylpyrrolidone, which were fabricated via electrospraying. Upon in vitro analysis, the formulation demonstrated superior skin permeation compared to free carvedilol for 24 h (*p* < 0.0001), attributed to the ethosomal formulation’s penetration ability. In vivo pharmacokinetic studies in rats showed that MNs-TDDP achieved a peak plasma concentration (C_max_) of 1749.44 ng/mL at 4 h (T_max_). The half-life (T_1/2_) extended to 4.77 h versus 3.63 h orally, indicating sustained release. Lastly, the animal studies proved that anti-hypertensive efficacy in spontaneously hypertensive rats was significantly improved, and serum nitric oxide levels were elevated. The safety aspects were well studied using histopathology (Figure 6).

Angiotensin II receptor blockers such as losartan and valsartan have also been synthesized as ethosome formulations. Researchers reported both drugs to have low bioavailability and a long partition coefficient. Since both have a low molecular weight, they are suitable candidates for transdermal drug delivery systems such as ethosomes. Research conducted by Vivek Dave et al. and Ahad et al. showed the efficacy of ethosomal and nanoethosomal transdermal delivery systems for anti-hypertensive drugs, losartan, and valsartan, respectively, revealing a significant improvement in sustained release and bioavailability [107,108]. Both studies optimized vesicular formulations with a particle size of 112.2 ± 1.3 nm, the zeta potential of −59 ± 0.8 mV, and 89.21 ± 2.82% entrapment efficiency, and VEGF-OPT-TTSs (valsartan ethosomal gel formulation-optimized-transdermal therapeutic systems) for valsartan with entrapment efficiency ranging from 63.66 ± 1.36% to 88.11 ± 2.65% and vesicle size of 63 ± 6.0 nm to 158 ± 7.0 nm, achieving superior pharmacokinetic profiles over oral administration. The in vitro release profiles were impressive, with the RL100-based losartan patch releasing 92.41 ± 1.98% of the drug over 72 h and valsartan nanoethosomes achieving a flux of up to 74.44 µg/cm^2^/h, highlighting their capacity for controlled, prolonged delivery. In vivo studies in rats further corroborated these findings: the losartan-loaded RL100 patch reduced blood pressure to 120.81 ± 2.56 mmHg after 48 h, surpassing oral losartan, while valsartan nanoethosomes increased AUC_0–48_ by 3.03-fold (165,192.0 ng/mL/h vs. 54,416.50 ng/mL/h) and extended the half-life to 39.85 h compared to 7.66 h for oral suspension. These results, underpinned by enhanced skin penetration (confirmed via confocal microscopy for valsartan) and stability, collectively position ethosomal/nanoethosomal patches as promising platforms for effective hypertension management, offering improved therapeutic outcomes through sustained and efficient drug delivery.

### 8.3. Management of Neurodegenerative Diseases

Neurodegenerative diseases are a group of conditions that progressively damage the nervous system, particularly the brain, spinal cord, and nerves [109]. This damage gradually leads to memory loss and coordination and cognitive decline [110]. Neurodegenerative diseases, the second leading cause of death globally in 2015, claim 80% of Alzheimer’s patients within one year, 55% within three years, and 60% within five years post-diagnosis [111]. Many types of neurodegenerative diseases exist, but Alzheimer’s disease, Parkinson’s disease, Huntington’s disease, etc., are very prominent and serious conditions. The treatment of such diseases requires effective molecules that can reach the brain. However, the blood–brain barrier (BBB) makes it tougher to reach those targets. In fact, the BBB serves the role of protecting the brain from unwanted chemicals by limiting permeability to most substances, even at the paracellular and transcellular levels. In general, essential molecules, such as nutrients, oxygen, carbon dioxide, etc., cross the BBB through passive diffusion [112]. However, in the case of small drug molecules, the BBB allows selective transport through carrier-mediated transport or active efflux transport; larger molecules have to choose receptor-mediated transport to cross the BBB [113,114]. Hence, crossing the BBB is always challenging, and thus, researchers prefer to adopt a carrier-mediated drug delivery method. Among the different nanocarriers used in brain delivery, lipid-based carriers are considered to be a favorable strategy [115]. Ethosomes have become an acceptable choice in this regard as they are more fluid and permeable in nature than the lipid bilayer. The large addition of ethanol makes it a negative surface charge, so the CNS delivery of the ethosomal formulation becomes practically possible by crossing the BBB [116].

Rasagiline is an irreversible inhibitor of MAO that is used for the management of idiopathic Parkinson’s disease. Navaneethan et al. prepared nanoethosomes and optimized them into a gel using the cold method [117]. They maintained the proportion of ethanol at 20–40% *v*/*v*, propylene glycol at 5–20% *v*/*v*, and soya lecithin at 2–4% *w*/*v* as independent variables, optimized via a D-optimal design. The entrapment efficiency was the key dependent variable. The optimum formulation (EF-8: 4% soya lecithin, 30% ethanol, 10% propylene glycol) achieved an entrapment efficiency of 91.11%, a vesicle size of 106.2 nm, and a zeta potential of −33.9 mV, indicating moderate stability. Scanning electron microscopy (SEM) confirmed a spherical bilayered structure. In vitro diffusion studies using phosphate buffer (pH 7.4) showed controlled drug release over 12 h, with the formulation exhibiting 90.55% cumulative drug release following a zero-order model and a non-Fickian diffusion mechanism (n = 1.2948). The optimized ethosomes were incorporated into a 2% *w*/*w* Carbopol 934 gel, which displayed a pH of 7.30, a viscosity of 2122.41 cP, a spreadability of 24.16 cm, and a drug content of 96.67%. A preformulation study for drug–excipient compatibility by FT-IR and DSC confirmed the purity of the drug, with no significant interaction between the drug and polymer observed. These findings suggest that Rasagiline-loaded nano ethosomal gel enhances bioavailability and brain targeting, offering a promising transdermal approach for Parkinson’s disease management.

Ligustrazine is an alkaloid isolated from a Chinese herb which has shown significant effects in treating Alzheimer’s disease. Earlier, some studies showed promising neuroprotective effects for treating Alzheimer’s disease-like pathologies. However, it has many disadvantages, like low bioavailability and the need for repeated administration. In order to overcome these challenges, Shi and colleagues have developed ethosomes, soft phospholipid vesicles with 30% ethanol, to enhance Ligustrazine’s transdermal delivery, overcoming limitations of oral/injection routes such as first-pass metabolism and frequent dosing [118]. The Ligustrazine ethosomal system showed a vesicle size of 146.3 ± 24.6 nm, an entrapment efficiency of 70.23 ± 1.20%, and stability with only a 2.2 ± 0.4% size increase after 4 weeks at 4 °C. In vitro studies using Franz-type diffusion cells and confocal laser scanning microscopy demonstrated superior skin permeation compared to an aqueous system. The ethosomal system achieved a steady-state permeation rate (J_s_) of 113.50 ± 12.6 μg/cm^2^/h versus 21.45 ± 4.1 μg/cm^2^/h for the aqueous system, with a lag time (T_lag_) of 2.0 h versus 2.3 h. The drug deposition in rat skin was 0.88 ± 0.12 μg/g (ethosomal) versus 0.51 ± 0.06 μg/g (aqueous). An in vivo pharmacodynamic evaluation was conducted in rats using a scopolamine-induced amnesia model. The Morris water maze test revealed reversed memory deficits, reducing escape latency from 104.76 ± 11.48 s to 30.48 ± 2.75 s. In addition, brain antioxidant enzyme activities and MDA (malondialdehyde) levels returned to normal, indicating oxidative stress mitigation. The results supported the use of the ethylmal formulation of ligustrazine for Alzheimer’s disease.

### 8.4. Management of Arthritis

Arthritis is a condition that exhibits symptoms like inflammation of the joints, leading to pain, stiffness, and sometimes swelling. It is considered a rare collection of many diseases rather than being termed as a single disease. Different types of arthritis exist, such as osteoarthritis, which comes with stiffness and inflammation of the joints when the protective cartilage that cushions the ends of the bones wears. From 1990 to 2021, global osteoarthritis cases in the working-age population surged by 116.16%, showing continuous upward trends in age-standardized incidence, prevalence, and DALY rates, particularly in high-income countries, with projections estimating 38,800,395 cases by 2040 [119]. Another type is rheumatoid arthritis, an autoimmune condition where the body attacks its own joint tissues [120]. Early diagnosis and pharmaceutical treatment are very important in this, as they will prevent the progression of the condition and improve patients’ quality of life. As we have seen in the earlier disease model, the treatment of arthritis also faces some serious challenges, like delayed drug delivery, decreased bioavailability, and so on [121]. For arthritis management, ethosomal formulations deliver anti-inflammatory drugs directly to the affected joints, achieving sustained local effects with minimal systemic exposure. Recent studies have explored ethosomes in multimodal strategies, such as combining naproxen-loaded ethosomes with microneedles to enhance penetration through inflamed skin, improving pain relief and joint mobility [122]. These targeted approaches offer precise drug delivery tailored to arthritis pathology, complementing conventional therapies.

**Figure 7:**
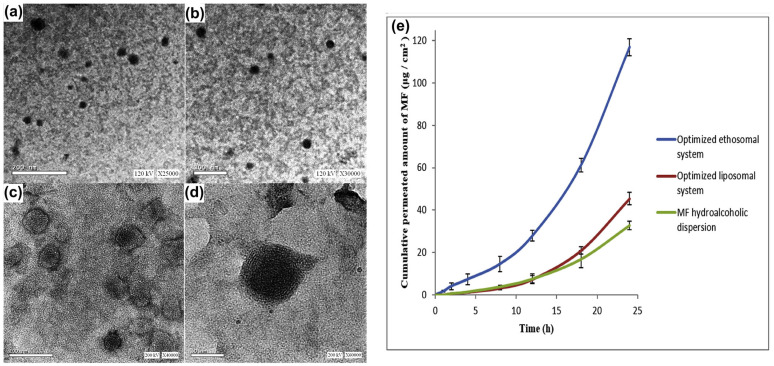
Mometasone furoate was formulated as an ethosome gel and evaluated for its anti-arthritic capacity in a rat model. The formulated vesicles were observed under transmission electron microscopy at different magnifications (**a**–**d**): (**a**) 25,000×, (**b**) 30,000×, (**c**) 40,000× and (**d**) 60,000×; (**e**) an ex vivo permeation profile of the formulation was observed at different mometasone-loaded ethosomes. Images were taken with permission from the reference [123]. Copyright 2020, Elsevier B.V.

Mometasone furoate is a corticosteroid used in the management of inflammatory conditions. Abdelbary and colleagues aimed to enhance the transdermal delivery of mometasone furoate for the treatment of inflammatory conditions like arthritis using an optimized ethosomal system [123]. Ethosomes, phospholipid vesicles with a high alcohol content, were developed to improve drug penetration through the skin’s stratum corneum. The formulation was optimized using a Box–Behnken design, evaluating four independent variables: Phospholipon 90G (PC90G, 1–3%), ethanol (25–45%), propylene glycol (PG, 0–20%), and the sonication time (0–60 min). These were assessed for their effects on entrapment efficiency, the vesicle size, the zeta potential, skin flux, and skin accumulation. The optimized mometasone furoate ethosomal formula achieved an entrapment efficiency of 68.44 ± 3.07%, a vesicle size of 62.87 ± 1.65 nm, a zeta potential of −15.92 ± 0.38 mV, a skin flux of 7.42 ± 0.13 μg/cm^2^/h, and a skin accumulation of 16.63 ± 1.65 μg/cm^2^/24 h. They compared the ethosomal formulation with drug-loaded liposomes and hydroalcoholic dispersion and found that the ethosomal drug formula exhibited superior permeation. Confocal laser scanning microscopy confirmed deeper penetration of the ethosomal formula into rat skin. Later, the ethosomal formulation was incorporated into a 1.5% Carbopol gel and tested in vivo against carrageenan-induced rat joint arthritis. After 20 days, the optimum ethosomal gel-treated rats showed complete histological recovery, outperforming commercial cream. This ethosomal gel showed excellent performance with stability for up to 120 days at 40 °C, highlighting ethanol’s role in enhancing skin flux and accumulation (Figure 7).

Skin permeation can be increased by adding physical methods like microneedling and ethosomal formulations. Researchers have successfully established these methods. Cui and colleagues attempted to increase the poor bioavailability of Paeoniflorin, a glycoside of plant origin that has shown anti-arthritic activity [124]. Paeoniflorin-loaded ethosomes were optimized using single-factor tests and an orthogonal experimental design, achieving an entrapment efficiency of 27.82 ± 1.56%, a particle size of 137.9 ± 7.57 nm, a polydispersity index of 0.120 ± 0.005, and a zeta potential of −0.74 ± 0.43 mV. Transmission electron microscopy confirmed a nearly spherical morphology. The optimal microneedle conditions were determined as 500 μm length, 3 N pressure, and 3 min application, balancing penetration enhancement and minimal skin damage. Ex vivo skin permeation studies compared a Paeoniflorin gucodide solution and an ethosome formulation with and without microneedle assistance. Ethosomes doubled Paeoniflorin penetration compared to the Paeoniflorin gucodide solution, while microneedling increased it 22-fold. However, combining ethosomes and microneedling showed no synergistic effect, likely due to Paeoniflorin’s water solubility and the limited ability of larger ethosomal particles (100 nm) to traverse microneedle-created pores compared to free Paeoniflorin molecules. Stability tests indicated that the ethosomes remained stable for 10 days, with the entrapment efficiency dropping slightly to 23.29 ± 1.39% by day 40. These findings suggest that while both methods enhance Paeoniflorin delivery, microneedles alone may be more effective for water-soluble drugs, offering insights for optimizing transdermal systems in anti-arthritic clinical applications.

### 8.5. Management of Cancer

Cancer is a group of diseases where abnormal cells grow uncontrollably, often forming tumors or leading to metastasis. The beginning of cancers starts from genetic mutations and environmental factors like smoking or radiation, disrupting the normal process of cell division. Many types of cancers exist, and they all require different approaches to cure or control them. Cancer can include surgery, radiation, and the most modern treatment, chemotherapy. The formulation of drugs for chemotherapy is often associated with nanotherapeutics, which make sure of its low dose to therapeutic ability, ease in delivery, and cost-effectiveness. Ethosome-based cancer therapeutics are available, especially for skin, lung, and breast cancers.

#### 8.5.1. Skin Cancer

Ethosomes are basically nanoformulations focused on the integumentary system, and hence, the most common application of ethosomes in cancer is in skin cancers. Melanoma skin cancer is the 17th most common cancer worldwide, with Europe seeing the highest overall cases and deaths. Australia and New Zealand have the highest incidence and mortality rates. Asia has a lower incidence but a higher mortality rate [125]. Skin cancers are divided into melanoma, non-melanoma cancers like basal cell carcinoma, and squamous cell carcinoma [126]. Melanoma is a cancer of the skin, where the cancer forms in the melanocytes. Ethosomes have emerged as a promising transdermal drug delivery system for melanoma treatment, as demonstrated by researchers. Ma et al. (2019) [127] developed a polyethylenimine and sodium cholate ethosome complex (7:3 ratio) that could co-deliver doxorubicin (DOX, 9 mg/kg) and curcumin (CUR, 21 mg/kg), achieving a particle size of approximately 100–200 nm, a positive ζ-potential of 21.8 ± 0.15 mV, and a tumor inhibition rate of 46.38% in C57BL/6 mice, significantly higher than single-drug ethosomes (*p* < 0.01). Uniquely modified ethosomes with polyethylenimine and sodium cholate exist for charge optimization [127]. Lin et al. formulated ethosomes with soybean lecithin, cholesterol, and 20–50% ethanol to co-load berberine chloride (BBR) and evodiamine (EVO), reporting an optimal size of 171 ± 7 nm, an entrapment efficiency of >90%, and epidermal deposition of 2.016 ± 0.237 mg/g berberine chloride and 0.196 ± 0.036 mg/g evodiamine, reducing B16 cell viability to 6.25 ± 0.17% at 18.6 µg/mL berberine chloride and 2.25 µg/mL evodiamine, while the control cells showed 87.59 ± 5.12% viability. The study emphasized sustained release (100% in 12 h) [128]. Both studies used ethanol (20–50%) to enhance skin penetration via stratum corneum fluidization, particle sizes generally below 319 nm, and in vitro/in vivo efficacy against melanoma or related skin cancers. They employed thin-layer techniques for preparation and demonstrated superior transdermal delivery over controls using Franz diffusion and mice models, with Ma et al. and Lin et al. focusing on multidrug synergy (DOX/CUR and BBR/EVO) in human cadaver skin.

The efficacy of ethosomes has been proven in non-melanoma cancers like basal cell carcinoma and squamous cell carcinoma. For instance, Paolino et al. have tested the efficacy of paclitaxel-loaded ethosomes in actinic keratoses and squamous cell carcinoma with the expectation of overcoming the hydrophobicity and systemic administration challenges of paclitaxel [129]. In order to enhance penetration, ethosomes were prepared. They formulated different types, of which one formulation (45% *w*/*v* ethanol, 1% *w*/*v* phospholipids) showed optimal properties: a small vesicle size (240.0 ± 61.48 nm), a narrow polydispersity index (0.244 ± 0.012), and high entrapment efficiency (82.0 ± 1.78%). Later, paclitaxel was incorporated with the ethosome vesicle, which showed a small vesicle size (240.0 ± 61.48 nm), a narrow polydispersity index (0.244 ± 0.012), and high entrapment efficiency (82.0 ± 1.78%). Transmission electron microscopy confirmed spherical, single-lamellar vesicles, while the zeta potential reached −49.5 mV with PTX incorporation, indicating stability. In an in vitro test, the paclitaxel formulation was found to be higher in dermal delivery, which is ~5-fold compared to the mixture and ~22-fold versus the suspension, demonstrating enhanced penetration to deeper skin layers. The anti-proliferative efficacy was tested on human squamous carcinoma cells. PTX-loaded ethosomes outperformed free PTX, achieving 90% cell mortality at 5 μM after 72 h and increased apoptosis from 36% (free PTX) to 61%. Empty ethosomes showed no significant toxicity, confirming carrier safety.

#### 8.5.2. Breast Cancer

**Figure 8:**
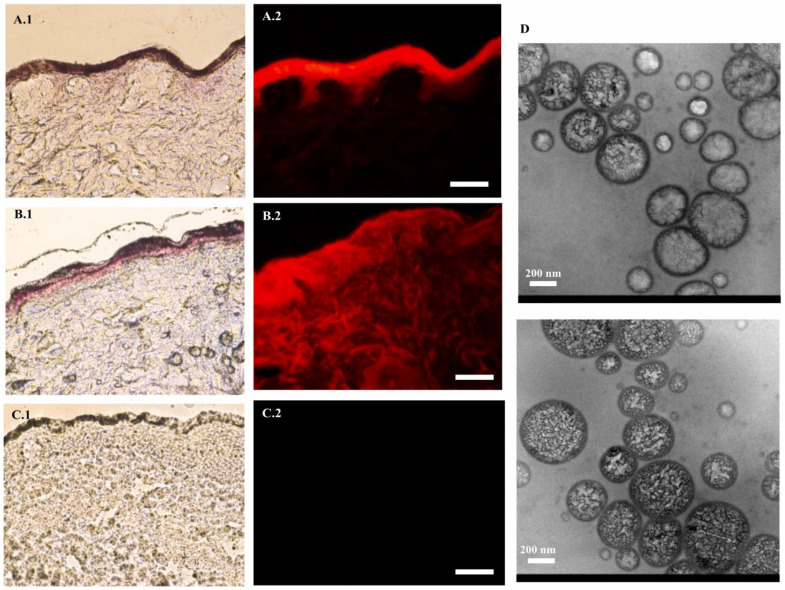
Fenretinide was formulated as an ethosome to act as a breast cancer chemopreventive treatment. The penetration and distribution of the fluorescent dye rhodamine B within skin tissue when incorporated into binary nanoformulations, as observed through various imaging techniques. Panels (**A.1**–**C.1**) present histological sections of skin visualized under halogen light, while panels (**A.2**–**C.2**) display fluorescence microscopy images (Bar = 100 µm) to assess dye distribution and autofluorescence. Panel (**D**) provides transmission electron microscopy images to characterize the morphology and size of the nanoformulations. The images were taken with permission from the reference [133]. Copyright 2021, Elsevier B.V.

Like all other solid cancers, breast cancer is also one of the most prevalent cancers. It is considered to be a cancer with high metastatic potential and is generally more drug-resistant [130]. Approximately one in three cancers in women is breast cancer, with around 15% of diagnosed cases resulting in death [131]. There are many challenges to the current treatment. For instance, inadequate specificity, the relatively low abundance of drugs in metastatic tissues, drug resistance, the physicochemical alteration of drugs, etc., are the primary issues [132]. Nanomedicines have always tried to promote treatment success in drugs that target the cancer cells in breast cancer. Studies on ethosome formulations have promising results. Apolinário et al. conducted research by a quality design approach to develop ethosomes for the topical delivery of fenretinide, a hydrophobic chemopreventive drug for breast cancer, addressing its poor solubility and bioavailability [133]. Ethosomes were selected for their ability to enhance drug solubility and skin penetration, leveraging ethanol injection for its simplicity and scalability over thin film hydration. The optimized binary ethosomes achieved a particle size of 378 ± 5 nm, a polydispersity index of 0.27 ± 0.00, and a zeta potential of 23 ± 6 mV, demonstrating stability and uniformity. Critical process parameters and critical material attributes were determined using a 2^3^ factorial Design of Experiments, targeting higher fenretinide incorporation and deeper skin delivery. Skin penetration studies using pig ear skin in Franz diffusion cells showed enhanced fenretinide delivery into deeper skin layers after 6 h, with quantification via HPLC. Fluorescence microscopy with rhodamine B confirmed the penetration of hydrophobic molecules like fenretinide. Later cytotoxicity studies have been carried out in MCF7 and MCF10A cells, in which fenretinide-loaded ethosomes showed significant cytotoxicity compared to normal MCF10A cells. Their study revealed the utilization of ethosomes in breast cancer drug delivery with accuracy and less toxicity, making it an excellent strategy for breast cancer treatment (Figure 8).

Ethosomes were also used to increase the therapeutic efficacy of natural products. There are many natural products that show significant effects against breast cancer, like black cumin, which is a rich source of biologically active thymoquinone. Nasri et al. (2021) introduce a novel ethosomal nanoparticle system designed for the controlled transdermal delivery of black cumin extract and doxorubicin to enhance their anticancer efficacy against breast cancer cells [134]. Black cumin, rich in thymoquinone, exhibits potent anticancer properties but suffers from poor solubility and bioavailability. In order to overcome this, ethosome soft vesicles composed of phospholipids, cholesterol, and ethanol were synthesized with optimized formulations (5% soy lecithin, 45% ethanol, and 1.5% cholesterol), achieving a vesicle size of ~20 nm, an entrapment efficiency of 98 ± 1%, and a zeta potential of −61 ± 2 mV. Stability tests at 4 °C over 90 days showed minimal changes. In vitro release profiles indicated a biphasic pattern, whereas ex vivo permeation using rat skin demonstrated fluxes of 9.6 for thymoquinone, with over 40% permeation after 24 h. Cytotoxicity assays on MCF-7 cells revealed IC50 values of 200 µg/mL for crude extract vs. the 96 µg/mL ethosome formulation, with improved cellular uptake. The study showed that natural products alone or together with chemotherapeutic agents should be used for breast cancer treatment using effective nanocarriers like ethosomes.

## 9. Safety Profile and Ethical Considerations of Ethosomes

One of the major constituents of ethosomes, phospholipids, such as phosphatidylcholine derived from soy or egg sources, are generally recognized as safe due to their biocompatibility and presence in biological membranes [135,136]. However, ethanol, typically present in concentrations ranging from 20 to 45% in ethosomal formulations, introduces potential risks. High ethanol content can cause skin irritation, dryness, or erythema, particularly with prolonged or repeated application [26]. In vitro studies have demonstrated that ethanol disrupts the lipid layer of the stratum corneum, which enhances permeability but may compromise the skin barrier function over time, potentially leading to localized toxicity [34,137]. Apart from local toxicity, systemic toxicity is another concern, although minimal, due to the transdermal route’s avoidance of first-pass metabolism. Nevertheless, long-term exposure data are limited, and chronic use may warrant further investigation, particularly for drugs that require sustained release. In vitro and in vivo studies have been conducted and agree with the safety of ethosomes. In vitro studies have shown that, generally, ethosomes are safer in dermal skin cultures. In addition, histological studies conducted with applied skin layers have shown no changes in the structure and thickness of the skin layer. There was no inflammatory cell infiltration observed. This trend was observed both in acute and chronic studies [138]. Paolino and colleagues conducted studies on human subjects to study the skin tolerability of ethosomes and found no erythema until 48 h of treatment [139]. It is worth noting that there were no significant post-marketing adverse drug reports from ethosome formulations [138].

Ethosomes have been investigated in clinical trials, particularly for their ability to enhance topical and transdermal drug delivery. Like all other pharmaceutical preparations, ethosomes and associated skin penetration enhancer trials need proper ethical guidelines and controls [140]. It is very important to have strict practice when dealing with vulnerable participants, using new research protocols, and using bioengineered skin surrogates. Patients must be fully apprised of potential risks, such as skin irritation or unknown long-term effects, balanced against therapeutic benefits [141]. Ethical concerns also extend to sourcing raw materials; for instance, phospholipids derived from animal sources may conflict with vegan or cultural preferences, requiring transparency in formulation disclosure. So far, there are no reports regarding unethical trials or research associated with ethosomes in animals and in human volunteers.

## 10. Future Perspectives and Conclusions

Substantial improvements have been made in the field of transdermal drug delivery based on ethosomes, which has established a solid platform for therapeutic applications in metabolic and chronic disorders. However, several possibilities remain underexplored, which present interesting possibilities for innovation and clinical applications. Primarily, ethosomal formulations can be enhanced further by adopting novel strategies. For instance, ongoing research has been carried out to optimize their stability, drug-loading capacity, and targeted delivery potential, even though the current ethosomes have superior skin permeation compared to conventional liposomes. The integration of stimuli-responsive components, such as pH- or temperature-sensitive lipids, is one promising method that has the potential to provide regulated drug release at specific disease sites, especially in chronic conditions like rheumatoid arthritis or diabetic wounds [90]. The development of hybrid ethosomes with adjustable ethanol concentrations that may be adjusted to specific medications or skin types is another area that is currently being explored. Advanced computational modelling and machine learning approaches could also accelerate such adjustable formulations and their optimization by predicting ethosome–skin interactions and drug release kinetics.

The combination effect of ethosomes with other cutting-edge technologies offers a transformative pathway to overcome current limitations in transdermal delivery. Combining ethosomes with nanocarriers, such as solid lipid nanoparticles or polymeric micelles, could enhance drug encapsulation efficiency and provide dual-release profiles. For instance, such combinations offer immediate release from ethosomes and sustained release from the secondary carrier, which is ideal for managing chronic diseases like hypertension or diabetes. Microneedling application, together with ethosomes, is being studied extensively these days. The stratum corneum bypass can be effectively achieved this way, in which future studies can be incorporated, such as dissolvable microneedles coated with ethosomal formulations that could deliver high drug payloads into deeper skin layers, improving systemic absorption for conditions requiring rapid onset, such as insulin delivery in diabetes management. Finally, ethosome formulations in metabolic and chronic diseases need clinical translation prospects. Although many preclinical studies have been conducted so far, their transfer from bench to bedside remains a major challenge. This requires large-scale clinical trials, particularly in metabolic and chronic diseases. Moreover, pharmacokinetic variabilities in different demographic populations also need to be thoroughly studied.

## 11. Conclusions

In conclusion, the prospects and future of ethosomes are remarkable. This review has elucidated the critical role of structural components such as phospholipids, ethanol, and water in ethosomal functionality. The therapeutic applications of ethosomes in metabolic and chronic diseases such as diabetes, neurodegenerative diseases, cancers, arthritis, and cardiovascular diseases underline their usefulness, offering a non-invasive alternative to conventional oral and injectable therapies. By improving drug bioavailability, reducing systemic side effects, and enhancing patient compliance, ethosomes address key challenges in managing these conditions. Despite these advancements, the aforementioned challenges need critical attention.

## Figures and Tables

**Table 1:** Summary of recent studies on developing and applying ethosomes for selected metabolic and chronic diseases.

Disease	Drug Delivered	Formulation	Vesicle Size	Entrapment Efficiency	In Vitro	In Vivo	Reference
Diabetes	Repaglinide	Dipalmitoyl phosphatidylcholine (DPPC), ethanol, and water	0.171–1.727 μm	56.75–92.28%	Permeation (63.64–96.96%).	An optimized ethosomal formulation, blended into a 1.5% Carbopol 940 gel, provided sustained antidiabetic activity in alloxan-induced diabetic rats compared to oral.	[66]
Diabetes	Linagliptin	Soya phospholipids, ethanol, and water	105.65 ± 0.22 nm	79.85 ± 0.41%	Drug release of 98.74% over 10 h.	-	[67]
Diabetes	Insulin	Lipid-based ionic liquid, ethanol, edge activators	300 nm	-	-	A nanovesicle formulations effectively lowered blood glucose levels in an animal model.	[68]
Diabetes	Glimepiride	Phospholipids, ethanol (10–50%), propylene glycol, and water	91–224 nm	42–78%	Ethosomes showed a transdermal flux 3 times higher than liposomes and 25 times higher than a plain drug solution.	-	[69]
Diabetes	Dapagliflozin	Cholesterol and soy-lecithin and water	103.46 ± 2.17 nm to 394.15 ± 1.06 nm	45–88%	Steady-state flux was 1.93 μg/cm^2^/h.	-	[70]
Hypertension	Carvedilol	Phospholipid, ethanol, water	46.75 ± 8.0 nm and 259.3 ± 8.02 nm	86% and 97%	In vitro releases of 70.25 ± 4.26% after 24 h.	-	[71]
Pulmonary arterial hypertension	Vardenafil hydrochloride	Phosphatidylcholine and ethanol	159.9 nm	81.3%	-	It offered longer systemic exposure and greater bioavailability than oral administration.	[72]
Hypertension	Nebivolol	-	73.50 ± 0.08 nm	86.46 ± 0.15%	-	Pharmacokinetic studies revealed that OED-TP1 had 7.9 times higher bioavailability than oral Nevilob^®^ tablets.	[73]
Hypertension	Carvedilol	Phospholipon 100 H, cholesterol, ethanol, and Transcutol P	201.55–398.55 nm	30.00–90.66%	-	A notable, gradual decrease (*p* < 0.01) in the mean arterial pressure of rats was observed.	[74]
Hypertension	Lercanidipine	Phospholipon 90G (PL90G), LER, and ethanol	210.87 and 400.57 nm	49.26 to 97.22%	-	The pharmacokinetics study showed a statistically significant (*p* < 0.05) threefold increase in LER bioavailability with transdermal nanoethosomal LER gel compared to oral LER suspension.	[75]
Alzheimer’s disease	Vinpocetine	-	50.57 ± 26.11 nm	97.51 ± 0.86%	-	The nanoethosomal formulation exhibited a significant increase in flux and entrapment efficiency compared to the control vinpocetine solution.	[76]
Alzheimer’s disease	Rivastigmine	Soya lecithin, ethanol	140.94 + 22.38 nm	62.87 ± 9.86%	The sustained effect was observed in this order: transethosomal suspension > ethosomal suspension > liposomal suspension > pure drug solution.	-	[77]
Parkinsonism	Rasagiline Mesylate	Ethanol, propylene glycol and phospholipids	256 nm	38%	The drug diffused through the nasal mucosa showed improved results, reaching 766 μg/cm^2^ within 6 h.	-	[78]
Parkinsonism	Ropinirole	Soya phosphatidyl choline and ethanol	320.45 nm	65.69 ± 3.5%	After 24 h, the optimized formulation showed a 64.8% release compared to the pure drug.	-	[79]
Parkinsonism	Rasagiline	Phosphatidylcholine and sodium deoxycholate	198.63 ± 34.98 nm	95.73 ± 0.09%	-	The optimal in situ gel demonstrated safety and biocompatibility on rats’ nasal mucosa, with improved brain bioavailability (131.17%).	[80]
Arthritis	Apremilast	Soy lecithin, ethanol, and apremilast	93 nm (E4) to 158 nm	93.56%	Higher drug level of 1.5% displayed a greater flux of 40.62 μg/cm^2^/h.	C_max_, plasma drug concentration 319.75 ± 35.28 ng/mL).	[21]
Arthritis	Naproxen	PL90G and cholesterol later crosslinked with Carbopol 934	251.1 ± 1.80 nm to 343.5 ± 3.23 nm	66%	Ethosomes-hydrogel exhibited a sustained release effect (>8 h).	It significantly reduced inflammation (84.63%) and paw volume (0.1935 ± 0.08 mL) in Albino Wistar rats with induced arthritis.	[81]
Arthritis	Aceclofenac	Ethanol 10–50% (*v*/*v*), lecithin 1–4% (*w*/*v*), propylene glycol 5–20% (*v*/*v*)	1.112 μm	91.06 ± 0.79%	After 24 h, the cumulative amount permeated from the optimized ethosomal system (ETP2) was 0.49 ± 0.032 mg/cm^2^, compared to 0.31 ± 0.036 mg/cm^2^ from Movon gel.	-	[82]
Arthritis	Capsaicin	Phospholipid (2%) mixture was prepared in 30% ethanol	295 and 271 nm	61.31 ± 3.45%	Ethosomal capsaicin vesicles showed 72.98 ± 2.84% capsaicin permeation with a flux of 15.20 ± 1.7 cm/h × 10^−3^ in a modified diffusion cell over 24 h.	Capsaicin-loaded ethosomes significantly reduced carrageenan-induced acute rat paw edema.	[83]
Skin cancer	5-aminolevulinic acid	-	<200 nm	8–66%	All formulations showed enhancements ranging from 11- to 15-fold compared to the control.	-	[84]
Skin cancer	Brucine	Cholesterol, phospholipid, ethanol	118 ± 1.5 nm to 218 ± 3.0 nm	50.2 ± 1.8% and 77 ± 1.2%	Over 6 h, the percentage of BRU released was 68.87 ± 3.9% from BRU-loaded gel, 50.87 ± 4.5% from BRU-loaded ethosomes, and 33.67 ± 3.92% from BRU-loaded ethosomal gel.	-	[85]
Skin cancer	Tocotrienol	Phospholipid, cholesterol, polysorbate 80, ethanol	64.9 ± 2.2 nm to 79.6 ± 3.9 nm	66.8 ± 1.9% and 68.5 ± 1.2%	After 48 h, the cumulative amount was 1.03 ± 0.24 mg/cm^2^ with a flux of 0.03 ± 0.01 mg/cm^2^/h. HaCat cells exhibited significantly higher cell viability compared to the pure drug.	The flux of gamma-T3 across the Strat-M^®^ and epidermal membrane was significantly greater than across full-thickness human skin.	[86]
Breast cancer	Retinoid fenretinide	Ethanol, propylene glycol, soya lecithin, water, and polysorbate 80 micelles	-	-	-	Oral administration kept plasma FENR levels below 10 ng/g in the first three hours, while MN administration delayed delivery, peaking at a maximum plasma concentration of 52 ng/g after 48 h.	[87]
Breast cancer	Raloxifene	-	109.2 ± 1.52 nm to 150.73 ± 4.10 nm	11.735 ± 0.19% to 62.65 ± 0.63	The transdermal flux of the optimized ethosome formulation was 22.14 ± 0.83 µg/mL/cm^2^, 21 times higher than that of conventional liposomes.	Rats exhibited a 157% higher bioavailability of RXL with the ethosomal formulation compared to oral administration.	[88]

## Data Availability

The data presented in this study are contained within this article.
